# TLR-Agonist Mediated Enhancement of Antibody-Dependent Effector Functions as Strategy For an HIV-1 Cure

**DOI:** 10.3389/fimmu.2021.704617

**Published:** 2021-09-23

**Authors:** Caroline Thue Hvilsom, Ole Schmeltz Søgaard

**Affiliations:** ^1^ Department of Clinical Medicine, Aarhus University, Aarhus, Denmark; ^2^ Department of Infectious Disease, Aarhus University Hospital, Aarhus, Denmark

**Keywords:** toll-like receptor agonists, broadly neutralizing antibodies, HIV-1 cure, effector mechanisms, ADCC, HIV reservoir, NK cells

## Abstract

**Background:**

The current treatment for HIV-1 is based on blocking various stages in the viral replication cycle using combination antiretroviral therapy (ART). Even though ART effectively controls the infection, it is not curative, and patients must therefore continue treatment life-long.

**Aim:**

Here we review recent literature investigating the single or combined effect of toll-like receptor (TLR) agonists and broadly neutralizing antibodies (bNAbs) with the objective to evaluate the evidence for this combination as a means towards an HIV-1 cure.

**Results:**

Multiple preclinical studies found significantly enhanced killing of HIV-1 infected cells by TLR agonist-induced innate immune activation or by Fc-mediated effector functions following bNAb administration. However, monotherapy with either agent did not lead to sustained HIV-1 remission in clinical trials among individuals on long-term ART. Notably, findings in non-human primates suggest that a combination of TLR agonists and bNAbs may be able to induce long-term remission after ART cessation and this approach is currently being further investigated in clinical trials.

**Conclusion:**

Preclinical findings show beneficial effects of either TLR agonist or bNAb administration for enhancing the elimination of HIV-1 infected cells. Further, TLR agonist-mediated stimulation of innate effector functions in combination with bNAbs may enhance antibody-dependent cellular cytotoxicity and non-human primate studies have shown promising results for this combination strategy. Factors such as immune exhaustion, proviral bNAb sensitivity and time of intervention might impact the clinical success.

## Introduction

Overwhelming progress has been made in preventing and treating human immunodeficiency virus 1 (HIV-1). Antiretroviral therapy (ART) can now effectively control viral replication, prevent the development of acquired immunodeficiency syndrome (AIDS), reduce risk of transmission and restore normal life expectancy (1). However, during the early phase of primary infection, HIV-1 integrates into the host genome establishing a latent reservoir that persists even after decades of ART. The reservoir persists primarily in long-lived infected CD4+ T cells containing transcriptionally silent, but replication competent provirus, that goes undetected by the host immune system and after ART cessation generally leads to rebound viremia (2–5). Life-long compliance to ART is therefore a requirement for virologic control until alternative treatment strategies can be developed enabling sustained ART-free virologic remission or complete eradication of the viral reservoir (6). One strategy aimed at eliminating the viral reservoir, hypothesize that the administration of latency reversing agents (LRAs), will reactivate HIV-1 transcription in latently infected cells, subsequently making cells sensitive to immune-mediated killing (7). However, thus far this strategy has not been effective in eradicating the HIV-1 reservoir.

During acute HIV-1 infection, an initial decline in plasma viremia occurs prior to the development of adaptive immunity, indicating that the innate immune system plays a potential role in controlling initial disease progression (8). It has been demonstrated that the priming of innate effector cells such as natural killer (NK) cells enhance their ability to recognize and eliminate infected cells, suggesting that agents which prime innate NK-mediated immunity can augment HIV-1 cure therapies (8). Toll-like receptor (TLR) agonists specifically TLR 7 and 9 agonists are mainly expressed on plasmacytoid dendritic cells (pDCs) and B cells and trigger not only innate immune functions but also plays a role in initiating the adaptive response. They are therefore favored in this strategy as they both function as LRAs and immune stimulatory compounds (9).

Broadly neutralizing antibodies (bNAbs) are able to engage both adaptive and innate immune responses. An important mechanism for eliminating infected cells is mediated by antibody-dependent effector functions such as antibody-dependent cellular cytotoxicity (ADCC). Some HIV-1 positive patients have been shown to develop broad serologic neutralization activity against conserved viral epitopes after a period of two to four years (10, 11). Multiple bNAbs have now been identified and are currently being evaluated for their ability to directly target HIV-1 infected envelope (Env) expressing cells, thus facilitating infected cell elimination through Fcγ receptor (FcγR)-dependent effector mechanisms.

Consequently, in this review we explore the current literature for evidence that stimulation of innate immune cells by TLR agonists in combination with bNAb administration may induce sustained remission in HIV-1 infected patients after ART cessation. We detail the current knowledge on the single and combined effect of TLR agonists 7 or 9 and bNAbs from preclinical and clinical studies. The present review focuses on the involved mechanisms including TLR agonist-induced innate immune activation, bNAb-binding to infected cells, ADCC capacity, viral reservoir size, and time to viral rebound during analytical treatment interruption (ATI).

## Immunomodulatory Properties of TLR7 and 9 Agonists

### *Ex Vivo/In Vitro* Studies

Four preclinical studies evaluated the ability of TLR7 or 9 agonists to enhance immune effector functions and HIV-1 transcription in latently infected cells (**Table 1**). A study by Bam et al. (12) investigated the ability of the TLR7 agonist GS-9620 to inhibit HIV-1 replication *in vitro* in both peripheral blood mononuclear cells (PBMCs) and isolated CD4+ T cells from donors infected with luciferase reporter HIV-1. GS-9620 showed antiviral activity in *ex vivo* HIV-1 infected PBMCs when pre-stimulated for 48 hours with GS-9620 prior to infection, however GS-9620 was inactive against HIV-1 in purified CD4+ T cells. An increase in the production of interferon-α (IFN-α) was observed in GS-9620-treated PBMCs and the co-treatment with IFN-α-blocking antibodies reversed the HIV-1 inhibitory effect. Additionally, IFN-α production was detected in isolated pDCs treated with GS-9620.

**Table 1:** Studies investigating TLR7 and 9 agonists as LRAs and immune stimulators.

Ref	Study design and cell type	TLR agonist tested	Endpoint	Results
***Ex vivo/in vitro* experiments**				
(12)	*In vitro*, pre-stimulation with GS-9620* for 48 hours prior to infection with luciferase reporter HIV-1 PBMCs from healthy donors** CD4+ T cells from healthy donors	TLR 7 agonist (GS-9620)	(1a) HIV-1 replication	(1a) Inhibition
			(1b) IFN-α-level in supernatant from PBMCs post GS-9620 stimulation	(1b) Increase
			(2) HIV-1 replication	(2) No inhibition
(13)	*Ex vivo* stimulation, HIV-1 RNA as latency reversal measure PMBCs from aviremic HIV-1 positive donors on ART CD4+ T cells from aviremic HIV-1 positive donors on ART Latent *in vitro* CD4+ T cells co-treated with GS-9620 and PGT121	TLR 7 agonist (GS-9620) AND bNAb (PGT121)	(1) Mean supernatant HIV-1 RNA-level	(1) 1.6 median increase (100 nM) compared to controls (P = 0.03)
			(2) Mean supernatant HIV-1 RNA-level	(2) No increase
			(3) GS-9620+PGT121	(3) GS-9620 enhanced PGT121-mediated killing of HIV+ CD4+ T cells
(14)	*In vitro* stimulation, p24 antigen or a GFP reporter gene as latency reversal measure CD4+ T cells from healthy donors infected with NL4.3 JLAT10.6 cells stimulated with supernatant from GS-9620 stimulated PBMCs	TLR 7 agonist (GS-9620, CL264)	(1) Latent HIV reactivation	(1) No significant difference in %p24+ cells
			(2) Latent HIV reactivation	(2) Significant difference in %GFP+ cells compared to controls (P < 0.001)
(15)	*Ex vivo* stimulation, p24 antigen (ELISA) as latency reversal measure PMBCs from aviremic HIV-1 positive donors on ART	TLR 9 agonist (MGN1703***)	(1) IFN-α-level in supernatant post MGN1703 stimulation	(1) Increase (P = 0.005)
			(2) usRNA-level in CD4+ T cells extracted post MGN1703 stimulation	(2) 1.4-fold median increase (P = 0.036) when 2-fold stimulation (6 nM to 6 μM)
**Non-human *in vivo studies***				
(16)	*In vivo*, NHP model 10 chronically SIVmac251-infected rhesus macaques on long-term ART: GS-9620 (n = 5) Control (n = 5) GS-9620 was administered every other week for a total of 10 doses	TLR 7 agonist (GS-9620)	(1) Plasma cytokine levels	(1) Increased levels of IFN-α and IL-6
		Intervention:		No increase in TNF-α, IFN-γ, IL-2
		0.15 mg/kg GS-9620 OR vehicle control	(2) Time to viral rebound	(2) No significant delay in time to viral rebound and all animals rebounded (> 50 SIV RNA copies/mL) within 2 weeks following ATI
		ATI: 1 month after last GS-9620 dose	(3) Safety and tolerability	(3) No adverse events or tolerability issues
(17)	*In vivo*, NHP model 21 chronically SIVmac251-infected rhesus macaques on long-term ART:	TLR 7 agonist (GS-9620, GS-986) Intervention:	STUDY 1 (1a) Peripheral immune cell activation (1b) Plasma cytokine levels	STUDY 1
	STUDY 1	STUDY 1	(2) Effect on plasma viremia	(1a) Transient CD69 upregulation on T, NK and B cells
	GS-986 (n = 4) Control (n = 6)	Dose escalation 0.1-0.3 mg/kg GS-986	(3) Time to viral rebound	(1b) Increased levels of IFN-α
		OR vehicle control		
	STUDY 2	STUDY 2		(2) Transient plasma viremia < 1000 SIV RNA copies/mL in 4/4 GS-986-treated animals
	GS-986 0.1 mg/kg (n = 3) GS-9620 0.05 mg/kg (n = 3) GS-9620 0.15 mg/kg (n = 3) Control (n = 2) 10 doses of GS-986 or GS-9620 every other week followed by a 3-month resting period and then another 9 doses	GS-986 0.1 mg/kg OR GS-9620 0.05 mg/kg OR GS-9620 0.15 mg/kg		
		OR vehicle control		(3) No significant delay in time to viral rebound and all animals rebounded (> 50 SIV RNA copies/mL) within 7-10 days following ATI
		ATI: 2 weeks after last TLR 7 agonist dose	STUDY 2	STUDY 2
			(1) Efficacy of increased number of TLR 7 agonist doses	(1) Two animals had no inducible viral reservoir and was aviremic for 700 days after ATI
(18)	*In vivo*, NHP model 6 chronically SIVmac239X-infected rhesus macaques on long-term ART: GS-9620 (n = 4) Control (n = 2) 12 doses of GS-9620 were administered approximately every other week followed by a resting period and then additional 5 doses	TLR 7 agonist (GS-9620)	(1a) Peripheral immune cell activation	(1a) No sign of CD4+ T cell activation
		Intervention:	(1b) Plasma cytokine levels	(1b) Transient increased levels of IFN-α and ISGs
		0.15 or 0.05 mg/kg GS-9620 OR vehicle control	(2) Effect on plasma viremia	(2) No increase
		ATI: approximately week 146 post infection	(3) Time to viral rebound	(3) All animals rebounded within 4 weeks following ATI
(19)	*In vivo*, NHP model 44 acutely SHIV-infected rhesus macaques on ART (start day 7):	TLR 7 agonist (GS-9620) AND bNAb (PGT121)	(1a) Peripheral immune cell activation	(1a) GS-9620 induced CD69 upregulation on CD4+ T and NK cells
		Intervention:		
	GS-9620 (n = 11) PGT121 (n = 11) GS-9620 AND PGT121 (n = 11) Control (n = 11)	GS-9620 0.15 mg/kg OR/AND PGT121 10 mg/kg OR vehicle control	(1b) Plasma cytokine level	(1b) Increased IFN-α-levels
		ATI: 16 weeks after last PGT121 and GS-9620 dose at week 130	(2) Viral rebound at day 196 after ATI	(2) 6/11 GS-9620+PGT121 treated animals rebounded 9/11 PGT121 treated animals rebounded 10/11 GS-9620 treated animals rebounded 11/11 sham treated animals rebounded
	10 doses of oral GS-9620 every 2 weeks from week 96-114 5 IV PGT121 infusions every 2 weeks from week 106-114		(3) Adoptive transfer and CD8+ T cell depletion	(3) 5/6 GS-9620+PGT121 treated animals remained suppressed > 6 months and did not reveal virus by adoptive transfer of PBMCs and LNMCs or CD8+ T cell depletion

(20)	*In vivo*, NHP model 16 acutely SHIV-infected rhesus macaques on ART (start day 14): GS-986 AND N6-LS AND PGT121 (n = 8) Control (n = 8)	TLR 7 agonist (GS-986) AND bNAbs (PGT121, N6-LS)	(1a) Peripheral immune cell activation	(1a) Not measured
		Intervention:		
	10 oral GS-986 doses every 2 weeks from week 14-32 5 IV PGT121+N6-LS infusions every 2 weeks from week 24-32	GS-986 0.1 mg/kg AND PGT121 10 mg/kg AND N6-LS 30 mg/kg OR vehicle control	(1b) Plasma cytokine level	(1b) GS-986 was associated with increased levels of IFN-α, TNF-α and IL-6 among other cytokines
		ATI: control arm (week 40), active arm (4 weeks after bNAb-plasma-level < 0.25 μg/mL)	(2) Time to viral rebound after ATI	(2) All 16 animals rebounded
				Median rebound time in control arm 3 weeks (2.5-5.5)
				Median rebound time in active arm 6 weeks (4.6-6.9) Equivalent to a 2-fold delay (P = 0.024)
**Clinical trials**				
(21)	Phase 1; randomized, double-blinded, placebo-controlled trial 48 chronically HIV-1 infected patients (plasma HIV-1 RNA < 50 copies/mL) on ART randomized:	TLR 7 agonist (GS-9620)	(1) NK cell activation	(1) Increase
		Intervention:		
	GS-9620 (n = 36) Placebo (n = 12) 6-10 doses every other week	6 dose escalation cohorts with GS-9620 1-12 mg OR matched placebo	(2a) Effect on plasma viremia	(2a) No significant change in plasma HIV-1 RNA
			(2b) Reduction in total CD4+ T cell viral DNA levels	(2b) No significant change
			(3) Safety and tolerability	(3) Generally safe and well tolerated with no severe adverse effects
(22)	Phase 1 + 2; randomized, exploratory trial 14 chronically HIV-1 infected patients (plasma HIV-1 RNA < 50 copies/mL) on ART:	TLR 9 agonist (MGN1703)	(1a) NK cell activation	(1a) Increase in activated NK cells compared to baseline (P = 0.001)
	MGN1703 (n = 12)	Intervention:60 mg MGN1703 OR placebo	(1b) Plasma cytokine level	(1b) Increased levels of IFN-α compared to baseline (P = 0.023)
	Optional ATI phase (n = 9) randomized 1:1: No MGN1703 + no ART MGN1703 + no ART	ATI: week 24	(2a) Reduction in total CD4+ T cell viral DNA levels	(2a) No significant change
			(2b) Time to viral rebound (viral load > 5000 copies/mL)	(2b) No significant difference in time to viral rebound between the two groups
	Doses 2 times weekly for 24 weeks		(3) Safety and tolerability	(3) Generally safe and well tolerated with limited adverse events

Most studies had multiple endpoints. The most relevant endpoints are outlined in this table; *TLR 7 agonist vesatolimod (GS-9620); **Healthy donors = healthy HIV-1 seronegative donors; ***TLR 9 agonist lefitolimod (MGN1703).

In line with these findings, Tsai et al. (13) demonstrated that GS-9620 induced latent HIV-1 RNA production in PBMCs from HIV-1 infected donors with a 1.6-fold increase compared to vehicle controls. No increase of HIV-1 RNA was detected when isolated CD4+ T cells were stimulated directly which is not surprising given that CD4+ T cells do not express TLR7 at physiologically active levels. When PBMCs were co-incubated with antibodies against IFN-α receptors, no increase in HIV-1 RNA levels were detected suggesting that the activation of integrated proviruses is at least partially induced by IFN-α.

Utilizing a primary cell model with CD4+ T cells latently infected with the replication-competent HIV-1 strain, NL4.3, Macedo et al. (14) tested latency reversal abilities of the two TLR7 agonists GS-9620 and CL264. Latency reversal was evaluated with a combination of readouts by measuring the induction of viral p24 Gag protein and by the surface downregulation of CD4 expression by the viral genes Nef and Vpu. Neither of the tested TLR7 agonists were observed to directly reactivate latent HIV in CD4+ T cells when compared to untreated controls. However, supernatant from PBMCs previously stimulated with GS-9620 effectively reactivated latent HIV in JLAT10.6 cells. This JLAT10.6 cell line harbors the full-length viral DNA that is transcriptionally silent in the absence of external stimuli and unresponsive to the TLR agonists used in this study. When stimulated with cytokines such as tumor necrosis factor-α (TNF-α), viral gene expression is activated, which can be measured by monitoring the expression of the reporter gene, GFP by flowcytometry (23). In order to identify the soluble factor(s) responsible for viral reactivation eight cytokines including IFN-α were tested. Contrary to Bam et al. (12) and Tsai et al. (13) only TNF-α was found to reactivate latent HIV even though increased levels of IFN-α were secreted from PBMCs stimulated with GS-9620. Additionally, supernatants treated with antibodies against TNF-α were unable to reactivate latent HIV in JLAT10.6 cells. Previous studies have indicated a cross-regulation between TNF-α and IFN-α (24), hereby suggesting that IFN-α is able to induce immune activation and subsequent production of TNF-α (14). In addition, Macedo et al. (14) found IFN-α secretion closely correlated with the ability of supernatants to reactivate latent HIV underlining the possibility of a synergistic effect on HIV reactivation with these two cytokines.

It is important to note, that Bam et al. (12) and Tsai et al. (13) used cells isolated from healthy donors and aviremic HIV-1 positive donors respectively, whereas Macedo et al. (14) used the JLAT10.6 cell line as target cells, which were clonally selected based on the ability to reactivate latent HIV by TNF-α stimulation (23). Despite discrepancies between applied methods and findings in the above studies, they all indicated that the latency reversing effect of GS-9620 on CD4+ T cells is being indirectly mediated by cytokine release from activation of mainly pDCs (9).

In an *ex vivo* model using PBMCs from aviremic HIV-1 infected donors, Offersen et al. (15) assessed the capacity of the TLR9 agonist MGN1703 to enhance immune effector functions as well as HIV-1 transcription. The study found a variable magnitude of response among PBMC donors, but a significant increase in IFN-α release from stimulated PBMCs (P = 0.005). CD4+ T cells isolated from MGN1703-incubated PBMCs also showed an enhanced transcription of HIV-1 unspliced RNA (usRNA) (P = 0.036).

As reviewed in Hornung et al. (25) and Adib-Conquy et al. (26), TLR7 and 9 are primarily expressed on DCs and B cells, and therefore the observed activation of NK and T cells is mediated indirectly *via* cytokine release and cell-to-cell interaction (15). In accordance with the findings above, this implies that the optimal *ex vivo* model for assessing TLR7 and 9 agonists is PBMCs instead of just isolated CD4+ T cells which will likely underestimate the potential *in vivo* effect.

### Non-Human Primate Studies

Bekerman et al. (16) conducted a study with 10 simian immunodeficiency virus (SIV)-infected rhesus macaques on long-term ART allocated to receive either GS-9620 or vehicle control every other week for a total of 10 doses. ART was continued during GS-9620 administration until analytical treatment interruption (ATI) was initiated one month after the last GS-9620 dose. Peripheral immune cell activation and plasma cytokine levels were measured 24 hours after each dose and significant increases in plasma IFN-α levels post-dosing were seen in GS-9620-treated animals compared to controls.

The study also evaluated the effect of GS-9620 on the viral reservoir and found no significant difference between GS-9620-treated animals and controls in total cell-associated viral DNA measured in PBMCs, lymph node mononuclear cells (LNMCs), and rectal biopsies. Additionally, there was no delay in time to viral rebound in GS-9620-treated animals compared to controls. All 10 animals rebounded within two weeks of starting the ATI.

Lim et al. (17) showed that GS-986 treatment in escalating doses was able to induce transient SIV plasma RNA blips < 1000 copies/mL in four out of four virally suppressed rhesus macaques on ART compared to zero out of six controls. Plasma viremia returned to baseline (< 50 SIV RNA copies/mL) within four to seven days after GS-986 dosing. T, NK and B cell activation, measured as an increase in CD69 expression, was detected within 24 to 48 hours after GS-986 treatment and returned to baseline before the next dose. An average reduction of 75% of SIV DNA in CD4+ T cells isolated from PBMCs, gastrointestinal mucosa mononuclear cells (GMMCs) and LNMCs was observed in GS-986-treated animals. Lastly, ART was discontinued to evaluate viral rebound kinetics, and viral rebound occurred in all animals within seven to 10 days with no significant difference in time to viral rebound being observed between GS-986-treated animals and controls. In a subsequent study, the same group tested the effect of an increased number of TLR 7 agonist doses and observed similar results. However, when assessing the viral reservoir, two TLR 7 agonist-treated animals were negative for SIV RNA induction in both PBMCs and LNMCs samples. The same two animals remained aviremic for more than 700 days after ART discontinuation whereas the remaining animals displayed rebound viremia within seven to 10 days. Adoptive transfer of PBMCs and LNMC from the aviremic animals did not induce *de novo* infection in naïve macaque recipients.

Del Prete et al. (18) were unable to reproduce results from Lim et al. (17) in a similar non-human primates (NHP) model. Six rhesus macaques were intravenously inoculated with SIVmac239X and treated with ART from day 13 post-infection. Seventy-five weeks after ART initiation animals were allocated to receive either 12 doses of GS-9620 (at 0.15 or 0.05 mg/kg) or vehicle control by oral gavage. ART was continued for a total of 144 weeks.

GS-9620 was shown to induce upregulation of multiple interferon-stimulated genes (ISGs) as well as significantly higher levels of plasma cytokines (IFN-α, IL-1RA, CXCL11) 24 hours post-dosing. The increase was transient and no significant difference between treatment groups was observed at before-dose time points. CD4+ T cells showed no increased activation following GS-9620 treatment, however 24 hours post-dosing GS-9620 induced significantly elevated CD38 expression (mean percentage of CD38+ increasing from 71% to 90%) as well as increased co-expression of CD38 and HLA-DR on CD8+ T cells indicating a significant CD8+ T cell activation. No measurable increase in plasma viremia was observed with SIV < 15 RNA copies/mL throughout the 12 doses of GS-9620 and no significant changes in transcriptional RNA levels in PBMCs, LNMCs or GMMCs was observed 24 or 48 hours post-dosing. All six animals rebounded within four weeks following ATI.

The reason for the conflicting findings between the two comparable NHP studies is not clear. In Del Prete et al. (18) animals were initiated on ART relatively early in infection (day 13) and were maintained on ART for 525 days prior to GS-9620 initiation. In comparison Lim et al. (17) initiated ART at day 65 post ART initiation and continued ART for 437 days before starting GS-986 treatment. It is possible that the viral reservoir established in animals from Del Prete et al. was too small to result in reactivated virus causing measurable changes of plasma viral loads. However, no consistent increase in viral transcriptional levels, measured as PBMC and tissue RNA/DNA ratios, were detected, which would be expected if sufficient viral reactivation had occurred. The timing of ART initiation and intervention could potentially affect the establishment of the viral reservoir causing the conflicting findings. NHPs from Lim et al. were infected using a low-dose intrarectal challenge with the SIVmac251 strain, whereas NHPs from Del Prete et al. were infected intravenously with the SIVmac239X strain. The different administration route as well as viral strain could also have contributed to the conflicting results. The relationship between ART initiation timing and duration, intervention timing and the established viral reservoir should be further investigated in future studies in NHPs.

Results from preclinical studies investigating the latency reversing effects of TLR7 or 9 agonists have showed promising evidence for a dual effect towards HIV-1 eradication. Partly by latency reversal in infected cells dependent on either a type I IFN production (12, 13, 15–17) or by TNF-α production (14) as well as enhanced Fc-mediated immune functions (13). However, as we discuss in the next section, clinical trials investigating the single effect of TLR agonists have not been able to show a sustained reduction in the size of the viral reservoir (21, 22).

### Clinical Trials

In a randomized, placebo-controlled trial conducted by Riddler et al. (21), 48 individuals were assigned to receive six to 10 doses of GS-9620 (1-12 mg) or placebo every other week in matched dose escalation cohorts. GS-9620 was generally safe and well tolerated with no adverse events leading to treatment discontinuation. GS-9620 did not induce plasma viremia and no significant differences in usRNA or total HIV-1 DNA in CD4+ T cells were observed between groups. GS-9620 treatment was associated with the induction of serum cytokines, however IFN-α mean levels were below the detection limit at most time points and generally only detectable for GS-9620 doses ≥ 6 mg. The study observed a dose-dependent induction of ISGs after GS-9620 administration of 4 mg or more with the greatest induction in the 8 mg group 24 hours post-dose, as well as an increase in the frequency of CD69+ NK cells at GS-9620 doses of 6 and 8 mg. Notably, the dose required to induce an ISG response (≥ 4 mg) was lower than that required for detection of serum IFN-α levels (≥ 6 mg). GS-9620 (Vesatolimod) is currently being evaluated for safety, tolerability and antiviral activity in HIV-1 viremic controllers on ART and subsequently during ATI (NCT03060447).

Vibholm et al. (22) conducted a 24-week exploratory study with 14 chronically infected HIV-1 patients on ART. Patients received 60 mg of the TLR 9 agonist MGN1703 two times weekly for 24 weeks followed by an optional ATI period. ATI participants were randomized to discontinue either ART and MGN1703 treatment simultaneously at week 24 or only ART while continuing MGN1703 treatment for an additional four weeks. The study found a systemic type I IFN response and enhanced activation of NK cells that was sustained for the duration of MGN1703 treatment. However, 24 weeks of MGN1703 treatment did not significantly reduce total HIV-1 DNA, usRNA or replication competent HIV-1 in CD4+ T cells in this cohort or delay time to viral rebound.

## Broadly Neutralizing Antibodies as Mediators of ADCC

We identified five preclinical studies evaluating the ability of different bNAbs to eliminate HIV-1 infected lymphocytes through NK-mediated ADCC. Profiling of bNAb-binding capacity, neutralization breadth and ADCC activity were evaluated using either cell lines or primary CD4+ T cells and either laboratory (lab)-adapted HIV-1 strains or primary viral isolates from HIV-1 positive patients. A summary of the *ex vivo, in vitro* and *in vivo* studies investigating ADCC-mediated elimination of HIV-1 infected cells by bNAbs and their main findings are shown in **Table 2**.

**Table 2:** Studies investigating ADCC elimination of HIV-1 infected cells by bNAbs.

Ref	Study design and cell type	Challenge virus	bNAbs tested	Endpoint	Results
**Ex vivo/in vitro experiments**					
(27)	Ex vivo, ADCC assay (AnV staining) CEM cells*, NK cells purified from healthy donors**	HIV-1 NL4.3	b12, VRC01, NIH45-46, 3BNC117, PG16, PGT121, 10-1074, 2G12	(1) Binding and ADCC activity against niCEM and byCEM cells	(1) All bNAbs that bound niCEM cells supported ADCC except 2G12 (P = 0.003)
					Env-level on byCEM cells was insufficient to cause robust ADCC
(28)	Ex vivo/in vitro, ADCC assay (L/D staining)	(1)HIV-1 NLAD8, NL4.3 (2)T/F viruses (3)Reactivated patient virus	VRC01, NIH45-46, 3BNC117, 12A12, PG16, PGT121, 10-1074, 8ANC195, 10E8, 4E10	(1) Identification of bNAbs that bind and kill lab-strain HIV-1 infected CEM cells (2) Binding and ADCC activity against various T/F virus CEM cells (3) Binding and ADCC activity against reactivated HIV-1 infected CD4+ T cells	(1) 8/12 bNAbs induced FcγRIII signalling in NLAD8-infected cells (similar for NL4.3, except V3-specific bNAbs) Most bNAbs triggered significant disappearance of infected cells (20-50% ↓Gag+ cells) (2) ↓Ability of bNAbs to bind and induce ADCC in cells infected with T/F viruses compared with lab strain (NLAD8) (3) Heterogeneity of reactivated cells associated with variable susceptibility to ADCC
	CEM cells, PBMCs from aviremic HIV-1 infected donors, NK cells purified from healthy donors		Controls: non-bNAbs (5-25) and (11-340), LALA control***		
(29)	Ex vivo, ADCC assay (Luciferase activity)	HIV-1 NL4.3 HIV-1_JR-FL_	b12, b6, VRC01, PGV04, 3BNC117, PG9, PG16, PGT126, PGT121, 10-1074, 2G12, 2F5, 4E10, 10E8	(1) ADCC activity against infected CEM cells (2) bNAb-binding and ADCC	(1) 12/14 bNAbs induced significant lysis of NL4.3-infected cells (except 2F5, 4E10)
	CEM cells, NK cell line expressing human CD16	SHIV_AD8-E0_			9/14 bNAbs induced significant lysis of HIV-1_JR-FL_ -infected cells (except MPER, 2G12 and b6)
					(2) bNAb-binding correlated with ADCC activity for all three viruses, strongest for HIV-1_JR-FL_ (P < 0.0001)
					Neutralization significantly correlated with ADCC for all tested viruses
(30)	In vitro, ADCC assay (HIV-1 Gag+) PBMCs and NK cells from healthy donors	HIV-1 NL4.3 10 primary HIV-1 isolates (2 clade A, 4 clade B, 2 clade C, 2 clade D)	VRC01, NIH45-46, 3BNC117, PG9, PGT145, PG16, PGT121, 2G12, 2F5, 4E10	(1) bNAb-binding against primary HIV-1 infected CD4+ T cells	(1) Significantly enhanced bNAb recognition of surface HIV-1 Env on CD4+ T cells with antibodies PG9, PGT145, PG16, 2G12 compared to control
			Control: F105	(2) ADCC activity against primary HIV-1 infected CD4+ T cells	High variability of antibody recognition of target cells infected with different clades
					(2) PG9 exhibited significant elimination of target cells ↓16.1% (P = 0.0312)
					Recognition of HIV-1 infected CD4+ T cells was significantly correlated with ADCC activity
(31)	Ex vivo, ADCC assay (L/D staining)	Reactivated patient virus from eight donors	VRC01, VRC07-523, 3BNC117, N6, PGT121, 2G12, 10-1074, PGDM1400, CAP256.VRC26.25, PG9, 10E8, 10E8v4-V5R-100cF, 2F5, 4E10	(1) bNAb-binding against reactivated reservoir virus infected CD4+ T cells (2) ADCC activity against reactivated reservoir virus infected CD4+ T cells	(1) CD4bs Abs (except VRC01) exhibited cell binding breath of 83-89% of viral isolates, MFI ratios 2-4
	PBMCs and NK cells from healthy donors, haNK cells****		Control: 4G2-Hu		V3 glycan antibodies exhibited cell binding breath of 42-75%, MFI > 5
					(2) Significant additional elimination of infected cells with the bNAb-addition
					Moderate correlation between bNAb-binding of infected cells and ADCC
**Non-human in vivo studies**					
(32)	In vivo, humanized mice model	Reactivated patient virus from one donor	10-1074	Time until viral rebound (undetectable viremia < 1000 copies/mL)	Untreated mice (12/12) displayed viremia after week 1
	Mice injected with PBMCs from a HIV-1 positive donor (n = 10)		Control: 10-1074-FcR^null^**		Viremia in mice treated with 10-1074 at week 1 (0/10, P < 0.0001), week 2 (0/9, P < 0.0001), week 3 (0/8, P = 0.005), week 4 (3/6, P = 0.036)
	Mice with PBMCs from a HIV-1 positive and negative donor (n = 12)				Mice treated with 10-1074-FcR^null^ week 2 (6/13)
(33)	In vivo, humanized mice model	HIV-1_YU2_	3BNC117, 10-1074	Disappearance of Gag+ cells	HIV-1_YU2_–infected cells reduced in mice treated with 3BNC117 (P < 0.005) and both 3BNC117 and 10-1074 (P < 0.001) compared to control
		HIV_2C1_	Isotype control		Similar results with primary strains (P < 0.05)
		HIV_2C5_			
		HIV_2D3_			
		HIV_2E5_			
**Clinical trials**					
(34)	Phase 2; randomized, double- blinded, placebo-controlled trial 19 chronically HIV-1 infected patients on ART:	HIV-1 subtype CRF01_AE, B, CRF01_AE and B coinfection, CRF01_AE/B/C recombinant	Intervention: 40 mg/kg VRC01 OR placebo 40 mg/kg 0.9% sodium chloride	(1) Proportion of participants with < 20 HIV-1 RNA copies/mL at 24 weeks after ATI (2) Safety and tolerability	(1) VRC01 treatment did not significantly impact the primary outcome 1 VRC01 treated individual remained virally suppressed for 42 weeks, all other participants rebounded (HIV-1 RNA ≥ 1000 copies/mL) and restarted ART before 24-week-endpoint
	• VRC01* (n = 14) • Placebo (n = 5) *One VRC01 participant did not complete ATI, due to severe urticaria				(2) VRC01 treatment was well tolerated with few adverse effects, except one case of severe urticaria
			ATI: 24 weeks		
(35)	Phase 1; randomized, double-blinded, placebo-controlled trial 40 chronically HIV-1 infected patients on ART:	HIV-1 subtype not stated	Intervention: 40 mg/kg VRC01 OR placebo 40 mg/kg 0.9% sodium chloride	(1) Change in cell-associated HIV-1 RNA/DNA ratio in total CD4+ T cells from baseline to 6 weeks (2) Proportion of CD+ T cells expressing HIV-1 RNA (3) Proportion of individuals with residual plasma viremia (4) Safety and tolerability	(1) No significant difference (2) No significant difference (3) No significant difference (4) Infusions were safe and well tolerated with few adverse events consistent with monoclonal antibody infusion reactions
	A (n = 20):				
	• VRC01 week 0 + 3 • Placebo week 6 + 9 (2) B (n = 20): • VRC01 week 6 + 9 • Placebo week 0 + 3 Follow-up: week 12-30				
(36)	Retrospective cohort study	HIV-1 subtype not stated	Natural bNAbs, no intervention	ADCC activity	Detected in most participants and increased over time (3 years)
	23 chronically HIV-1 infected adults • 13 bNAb-positive • 10 non-bNAb positive Longitudinal sample collection and 3-year follow-up				Six-month post infected significant difference prior to development of neutralization breadth
					At end 3-year follow-up no significant difference

Most studies had multiple endpoints. The most relevant endpoints are outlined in this table; *CEM.NKR.CCR5 cells; **Healthy donors = healthy HIV-1 seronegative donors; ***Mutated Fc region; ****Enhanced for ADCC.

### *Ex Vivo/In Vitro* Studies

There is no consensus assay for evaluating ADCC activity in an HIV setting and the lack of a standardized method to determine the viability of infected target cells makes it challenging to directly compare results across studies. Dupuy et al. (27) designed a novel flow-based measurement of ADCC using annexin V (AnV) staining of target cells and compared this method with previous ADCC assays. The AnV-assay was significantly more sensitive for the detection of target cell elimination, detecting both dying and dead cells, when compared with the Live/Dead (L/D) staining method, that only detected dead cells. This suggests that studies using the L/D method may underestimate the extent of ADCC. Furthermore, the AnV-assay is specific for target cells expressing HIV-1 Env and was shown to be more sensitive than the ADCC-GranToxi-Lux (ADCC-GTL) assay, which indirectly detects apoptosis by measuring the delivery of granzyme B to target cells (37). The rapid fluorometric ADCC (RFADCC) assay has been widely used to measure ADCC, however this assay quantifies membrane exchange between target and effector cells rather than ADCC activity directly (38, 39). The ADCC-AnV assay measures ADCC by identifying and quantifying dying and dead cells with a sensitivity superior to previous methods, hereby making it an advantageous method for evaluating ADCC activity in future studies.

Applying this novel method of ADCC detection, Dupuy et al. (27) conducted a comprehensive study using CEM cells infected with the laboratory HIV-1 strain, NL4.3. Antibody binding capacity and ADCC activity of a panel of bNAbs were evaluated using the AnV staining method against both p24 confirmed newly infected (niCEM) cells, bystander (byCEM) cells with shedded gp120 and non-infected (nCEM) cells, to evaluate the bNAb activity towards genuinely infected cells *versus* bystander cells (40). ADCC activity was found to be associated with binding capacity for all tested bNAbs with the exception of 2G12 that bound the HIV-1 Env protein on both niCEM and byCEM with a high mean fluorescence intensity (MFI) without inducing ADCC. 2G12 is suggested to have an unusual domain-swapped configuration which may affect the ability of this bNAb to interact with Fc receptors and support ADCC (41, 42). This association was supported by a significant correlation between frequency and MFI of bNAb-binding to Env and the frequency of AnV+ niCEM cells generated in the ADCC-AnV assay (P = 0.0003). The study importantly differentiates between ADCC detected towards genuinely HIV-infected cells and HIV-uninfected bystander cells when conducting assays, otherwise risking an overestimation of the ability of antibodies to mediate ADCC.

Bruel et al. (28) investigated antibody binding and ADCC activity of 10 bNAbs against CEM cells infected with the laboratory strains NLAD8 or NL4.3 and Transmitted/Founder (T/F) viruses. In line with the results from Dupuy et al. (27), most antibodies triggered a significant elimination of CEM cells infected with the lab-adapted HIV-1 strains while also exhibiting a significant correlation between binding potency and NK-mediated killing activity. When the panel of bNAbs was evaluated against CEM cells infected with T/F viruses, bNAbs had significantly lower binding and a reduced killing activity against these target cells when compared to NLAD8 or NL4.3 infected cells (28).

Furthermore, the study evaluated binding and ADCC activity of bNAbs against reactivated CD4+ T cells isolated from aviremic HIV-1 positive patients on ART and found considerable heterogeneity in the level of Env expressed on reactivated cells associated with a variable susceptibility to ADCC. Similarly, von Bredow et al. (29) observed that NL4.3-infected cells were generally more sensitive to ADCC, both in terms of the number of ADCC active antibodies and the magnitude of responses when compared to cells infected with a primary HIV-1 strain.

ADCC-mediated elimination of infected cells relies on interaction between the Fc region of bNAbs and the FcγR on effector cells. This interaction can be demonstrated by introducing mutations in Leu234Ala and Leu235Ala (commonly called LALA mutations) which more or less eliminates human cell FcγRIII binding (43). This indicates that the difference in killing efficacy may be caused by changes in antibody binding and the availability of the Fc region when bound to infected cells (28).

Some uncertainty persists with regards to bNAbs binding of free virus *versus* cell-bound HIV Env. Mujib et al. (30) observed a lack of binding of primary HIV-1 infected CD4+ T cells with the bNAbs VRCO1, 3BNC117 and NIH45-46 suggesting a different conformation of the HIV-1 Env on the surface of infected CD4+ T cells compared with cell-free viruses that these antibodies have been demonstrated to efficiently neutralize (28, 29). Moreover, von Bredow et al. (29) described instances of ADCC in the absence of detectable neutralization.

Multiple *in vitro/ex vivo* studies investigating both laboratory and primary viral isolates observed a difference in susceptibility to ADCC (28–30). In general, studies report clinical viral isolates to be less sensitive to bNAbs than laboratory strains (44, 45). These observations underline the limitation in directly correlating results from experiments exclusively using laboratory strains to the physiological conditions *in vivo* (46). In addition, the use of primary CD4+ T cells as target cells in assays instead of cell lines has been suggested to best represent the availability for antibody binding *in vivo* as these cells express Env on their surface in its native conformation (30). Likewise, the use of primary NK cells best represents the *in vivo* effect of ADCC (30).

When comparing ADCC assays performed with 36 viral strains isolated from eight patients undergoing ART, Ren et al. (31) found both inter- and intraindividual variability. This underscores the importance of tailoring possible future antibody treatment strategies to match the patient’s distinct viral reservoir and the limitation of using any single antibody as a treatment.

Mujib et al. (30) investigated 11 different HIV-1 strains representing clades (A-D) and found a significant interclade variation of binding capacity. This suggests that results from studies only investigating one HIV-1 clade, such as the study by Ren et al. (31) with a predominant North American clade B infected cohort, are not directly comparable to populations in which infection with non-B clades dominate.

Most *in vitro/ex vivo* studies found that bNAbs significantly enhanced the elimination of infected cells by ADCC and that ADCC activity correlated with binding and neutralization for each virus tested. The findings indicate that these functions overlap when testing antibodies with matched IgG1 Fc domains (27–30). However, Ren et al. (31) found the correlation between infected cell binding and ADCC to be weak, underlining that binding should not be considered the only measure of ADCC activity.

ADCC is considered primarily to be mediated by NK cells *via* FcγRIIIa, even though other immune cells, e.g., monocytes and granulocytes also express Fcγ receptors capable of ADCC (39, 47). An increasing amount of literature suggests that monocytes can function as potential mediators of ADCC in the context of HIV infection with similar levels of infected cell killing compared with NK cells (48–50).

ADCC has mainly been studied using IgG antibodies targeting gp120 of the HIV-1 Env, rendering the effects of gp41 specific antibodies as well as IgA isotype antibodies elusive. A study by Duchemin et al. (51) found that 2F5-IgA - a gp41 specific bNAb modified from 2F5-IgG - was able to engage FcαRI on human monocyte effector cells and enhance ADCC mediated killing of HIV-1 clade A and B infected target cells. When 2F5-IgA was combined with the IgG isotype bNAbs, 2F5-IgG or 10E8-IgG enhanced target cell lysis by ADCC was observed. However, other studies, including the RV144 clinical trial, have described vaccine-induced gp120-specific IgA to block gp120-specific IgG-binding sites, hereby attenuating IgG-mediated ADCC (52, 53). Tomaras et al. used NK cells that do not express the FcαRI which is essential for IgA-mediated ADCC and were therefore unable to induce IgA-mediated ADCC despite of efficient IgA-binding to infected cells, thus blocking IgG-binding and ADCC activity in this study.

Future studies should consider other immune cells, such as monocytes as effector cells in ADCC studies as well as further elucidate the effect of IgA isotype bNAbs on ADCC efficacy in relation to IgG.

### *In Vivo* Studies

A study by Flerin et al. (32) observed a significantly delayed time to viral rebound after the administration of the bNAb 10-1074 compared to treatment with a control-bNAb in immunodeficient mice that were intra-splenically injected with PBMCs from a long-term ART suppressed HIV-1 infected donor. This finding suggests that the 10-1074 treatment reduced the population of replication-competent latently infected cells through Fc-mediated effector mechanisms. Lu et al. (33) investigated the effect of the bNAb 3BNC117 alone or in combination with 10-1074 in a humanized mice model and also found a significant reduction of the number of HIV-1 infected cells (P < 0.005).

### Clinical Trials

Crowell et al. (34) conducted a randomized, placebo-controlled trial evaluating the effect of VRC01 (bNAb) administration on time to viral rebound during ATI in adults who began ART during acute HIV infection. 23 HIV-1 infected patients were enrolled of which 18 received at least one infusion of VRC01 or placebo every three weeks for up to 24 weeks during ATI. One VRC01 patient achieved the primary endpoint of less than 20 HIV-1 RNA copies/mL at 24 weeks after ATI and remained virally suppressed for 42 weeks. All other patients experienced viral rebound and restarted ART before the 24-week-endpoint highlighting both the limitations of single bNAb treatment and perhaps the potency and limited breath of VRC01.

Riddler et al. (35) reported findings from a randomized, placebo-controlled trial including 40 chronically HIV-1 infected individuals on ART. In two study-arms, VRC01 or placebo were administered at entry and week three and alternating the treatments at week six and nine. In agreement with findings from Crowell et al., VRC01 did not have a significant impact on neither HIV-1 RNA or DNA levels, cellular HIV-1 RNA/DNA ratio, HIV-1 plasma viremia or stimulated virus production from total CD4+ T cells.

A retrospective cohort study performed by Richardson et al. (36) evaluated samples collected over a period of three years from HIV-1 infected participants in the CAPRISA acute infection cohort. Thirteen individuals who had developed bNAbs were matched with 10 non-bNAb-positive controls with similar viral levels. ADCC was detected in the majority of participants early in infection and increased over time throughout the three-year follow-up period. Six-months post-infection, the authors found significantly higher levels of antibody-dependent complement deposition (ADCD) and cellular trogocytosis (ADCT) among bNAb compared to non-bNAb-positive individuals that correlated with antibody binding to C1q and FcγRIIa. In relation to neutralization breadth, ADCC activity did not differ between the two groups and no difference was seen at the three-year follow-up endpoint (33, 36).

Although some doubt has been raised regarding the importance of Fc-mediated functions in one study that found Fc dependent functions partially redundant when testing the bNAb PGT121 (54), several other studies have demonstrated enhanced ADCC elimination of HIV-1 infected cells. However, the very low levels of HIV-1 Env expression on latently infected cells during long-term ART may limit ADCC and thereby the therapeutic effect of bNAbs. Studies in HIV-1 infected patients (34–36) have indicated that monotherapy with the bNAbs VRC01, 3BNC117 does not reduce the size of the latent reservoir when patients are on concomitant ART. A few bNAb participants were reported to have achieved long-term control of viremia during subsequent ATI but the clinical trials were limited by small population sizes and lack of a control group. Thus, there is not enough evidence to support that antibody-dependent effector mechanisms induced by monotherapy with bNAbs leads to post-treatment control when ART is stopped.

## Combining bNAbs in HIV-1 Cure

The effects of bNAbs can be mediated both by neutralization and by Fc-mediated effector functions such as ADCC. Combining two or more bNAbs, targeting multiple epitopes on the HIV Env, may provide broader coverage and passive protection against circulating HIV strains compared to a single antibody. By extension it can be proposed that similar combinations of bNAbs in relation to ADCC will ensure the broadest recognition and elimination of infected cells. Cohen et al. (55) investigated safety and pharmacokinetics of combined infusions with the bNAbs 3BNC117 and 10-1074 in healthy adults. Participants were randomized to receive either one infusion of 3BNC117 immediately followed by 10-1074 at 10 mg/kg, three infusions of 3BNC117 followed by 10-1074 at 3 mg/kg or 10 mg/kg every eight weeks or placebo. Antibody concentrations were measured by two different methods, ELISA and the TZM-bl neutralization assay, with general concordance between the two. The mean elimination half-lives for 3BNC117 and 10-1074 were found to be 16.4 ± 4.6 days and 23.0 ± 5.4 days, respectively and no serious adverse events were observed. Of note, no association between elimination half-life and dose level administered, or single or repeated doses for either antibody was observed (P > 0.05). 3BNC117 and 10-1074 have previously been shown to suppress viremia in HIV-1 infected individuals, when administered alone and as single or repeated doses ranging from 1 to 30 mg/kg (56, 57). Cohen et al. compared ELISA data from HIV-uninfected individuals from these two studies receiving either 3BNC117 or 10-1074 with data of participants from their current study receiving the combination. Single or combined administration of the antibodies did not significantly affect the elimination half-life of either antibody, 3BNC117 (mean half-life: single 19.18 ± 7.08 days *vs.* combined 16.4 ± 4.6 days, P = 0.484) and 10-1074 (mean half-life: single 26.7 ± 4.5 days *vs.* combined 23.0 ± 5.4 days, P = 0.398).

Evaluating the effects of the combination of 3BNC117 and 10-1074 on maintaining HIV-1 suppression during ATI, Mendoza et al. (58) enrolled 15 HIV-1 infected participants, retrospectively clade B determined, to receive three infusions of 30 mg/kg each of 3BNC117 and 10-1074 at an interval of three weeks beginning two days before ATI. Time to viral rebound was defined as two consecutive viral loads of > 200 HIV-1 RNA copies/mL. Four participants were not included in the efficacy analysis as they developed viral loads of > 20 HIV-1 RNA copies/mL before or at the time of the first bNAb-infusion. Infusions were generally well tolerated, and no serious adverse effects were reported. As in HIV-uninfected individuals (55–57), the elimination half-lives of 3BNC117 and 10-1074 administered in combination, 17.6 days and 23.2 days respectively, were similar to those observed in previous studies when each antibody was administered alone underlining that the pharmacokinetics of 3BNC117 and 10-1074 are not altered when administered in combination. The combination of 3BNC117 and 10-1074 delayed viral rebound for a median of 21 weeks among the 11 participants who maintained viral suppression (< 20 HIV-1 RNA copies/mL) during the screening period and at day 0. Historical controls who received monotherapy with two or four 3BNC117 infusions experienced a delay in viral rebound of 6.7 and 9.9 weeks respectively (59).

Circulating HIV-1 variants present during active infection complicate viral control in viremic individuals compared to sustaining viral suppression in ART-treated individuals during ATI. In seven viremic clade B HIV-1-infected individuals (mean viral load > 11,494 HIV-1 RNA copies/mL), 3BNC117 and 10-1074 were administered in combination at either a single infusion of 30 mg/kg or three infusions every two weeks (60). When compared to a single infusion with either antibody 3BNC117 (56) or 10-1074 (57), the combination showed prolonged viral suppression (P = 0.00018). Viremia remained significantly reduced until day 86 with an average drop in viral load for all viremic individuals of 1.65 log_10_ copies/mL.

The combination of 3BNC117 and 10-1074 induce sufficient viral suppression in sensitive individuals suggesting that viral replication can be limited by this combination. Particularly, viral rebound was not observed when the concentrations of both antibodies were above 10 μg/mL (58, 60).

Tuyishime et al. (61) analyzed the potency and breadth of ADCC-competent bNAb and non-neutralizing antibody (nNAb) combinations in an *in vitro* system using concentrations of ≤ 1μg/mL. In order to best replicate the effects of the combinations *in vivo*, an autologous *in vitro* system with CD4+ T cells, infected with latent reservoir HIV-1 viruses (LRVs) from 10 chronically infected individuals, as target cells and autologous purified NK cells as effector cells was used. All LRVs were classified as clade B. Different combinations of bNAbs targeting non-overlapping epitopes expressed on the HIV-1 Env spike protein as well as on the surface of infected cells were evaluated as well as the nNAb A32. Within two hours the combination of three antibodies mediated more than 30% killing of infected cells, with the combination of the nNAb A32 (C1C2) and the two bNAbs DH511.2K3 (MPER) and PGT121 (V3) being most effective. The same combination was able to eliminate reactivated latently HIV-1 infected cells in an *ex vivo* quantitative viral outgrowth assay.

The combination of two or more anti-HIV-1-antibodies targeting multiple viral epitopes may be able to overcome viral resistance and increase the efficacy of virus neutralization and ADCC-mediated elimination of latently infected cells when administered in combination with LRAs. In a phase 1 trial 3BNC117 combined with 10-1074 administered over seven infusions at a dose of 30 mg/kg is currently being evaluated for safety and antiretroviral activity in HIV-1 infected individuals on ART during ATI (NCT03526848).

## Modified bNAbs in HIV-1 Cure

The plasma half-life of monoclonal IgG antibodies is closely related to the degree with which the antibody binds the neonatal Fc-receptor. Modifications to the constant (Fc) region on antibodies can therefore be made to alter the half-life of antibodies. In a phase 1 dose-escalation study safety and pharmacokinetics were evaluated for VRC01LS - a modified version of the CD4-binding bNAb VRC01 - in HIV-negative adults (62). VRC01LS is modified with two amino acid changes in the Fc region (M428L and N434S) in order to obtain a longer serum half-life. The modified VRC01LS demonstrated a four-fold increase in elimination half-life (t_1/2_ = 66 ± 24 days) compared to historical data for VRC01 (t_1/2_ = 15 days) (63). The LS mutation does not impact binding to the FcγRIII and thus retains the ability to induce Fc-mediated ADCC (64, 65). Other studies in NHPs confirm these findings (66), indicating that the LS mutation could potentially provide improved efficacy of neutralizing antibodies with less frequent dosing and a lower dose regimen. Additional phase 1 trials are currently evaluating safety, pharmacokinetics and antiviral activity of VRC01LS in HIV-1 infected or uninfected individuals (NCT02797171, NCT02840474, NCT02256631) as well as the combination of 3BNC117-LS and 10-1074-LS (NCT04250636).

Technological advances have also made it possible to modify the Fc region of monoclonal antibodies to enhance specific antibody-dependent effector functions. The Fc region can be engineered to have higher affinity for human FcγRIII which has been shown to increase ADCC. In fact, GS-9722 - a Fc-modified version of the V3-loop binding bNAb PGT121 - has been developed specifically for HIV cure purposes (67). The optimization process identified mutations that enhanced binding to activating FcγRs as well as the neonatal Fc-receptor. The resulting antibody demonstrated significantly enhanced killing of HIV infected CD4+ T-cells by primary NK cells isolated from human donors (mean values: Emax = 71%, EC50 = 0.23 μg/mL) compared to PGT121 (mean values: Emax = 11%, EC50 = 3.4 μg/mL). The Fab mutations had minimal impact on neutralization breadth or potency when tested against a panel of clade B viruses. GS-9722 (Elipovimab) is now in phase 1 clinical testing.

Multiple virological assays have been developed to detect levels of reactivable virus in latent cells on ART, in order to evaluate the success of an intervention in clinical trials but the correlation of these reservoir measures with time to viral rebound is not very strong. Time to viral rebound remains the best and clinically most relevant indicator of intervention efficacy. Interestingly, Offersen et al. (68) were able to show that HIV-1 specific antibody Fc N-galactosylation (glycosylation) is associated with viral rebound after ATI across three independent HIV cohorts. The mechanistical involvement of these glycosylated gp120 Env specific antibodies in viral control remains unclear and only antibody-dependent neutrophil phagocytosis (ADNP) and ADCD were linked to rebound time in this study. Although NK-mediated ADCC was not found to predict viral rebound time, others have pointed to NK cell activation as an important predictor of time to viral rebound in NHPs in a study that tested a combined LRA and bNAbs HIV cure approach (19). In addition to the emerging association of antibody glycosylation changes with reservoir size across a different independent cohort (69), virus-specific antibody glycosylation may pose an advantageous and simple strategy to track both reservoir size and reactivation.

## Combinations of bNAbs and TLR Agonists in HIV-1 Cure

**Figure 1:**
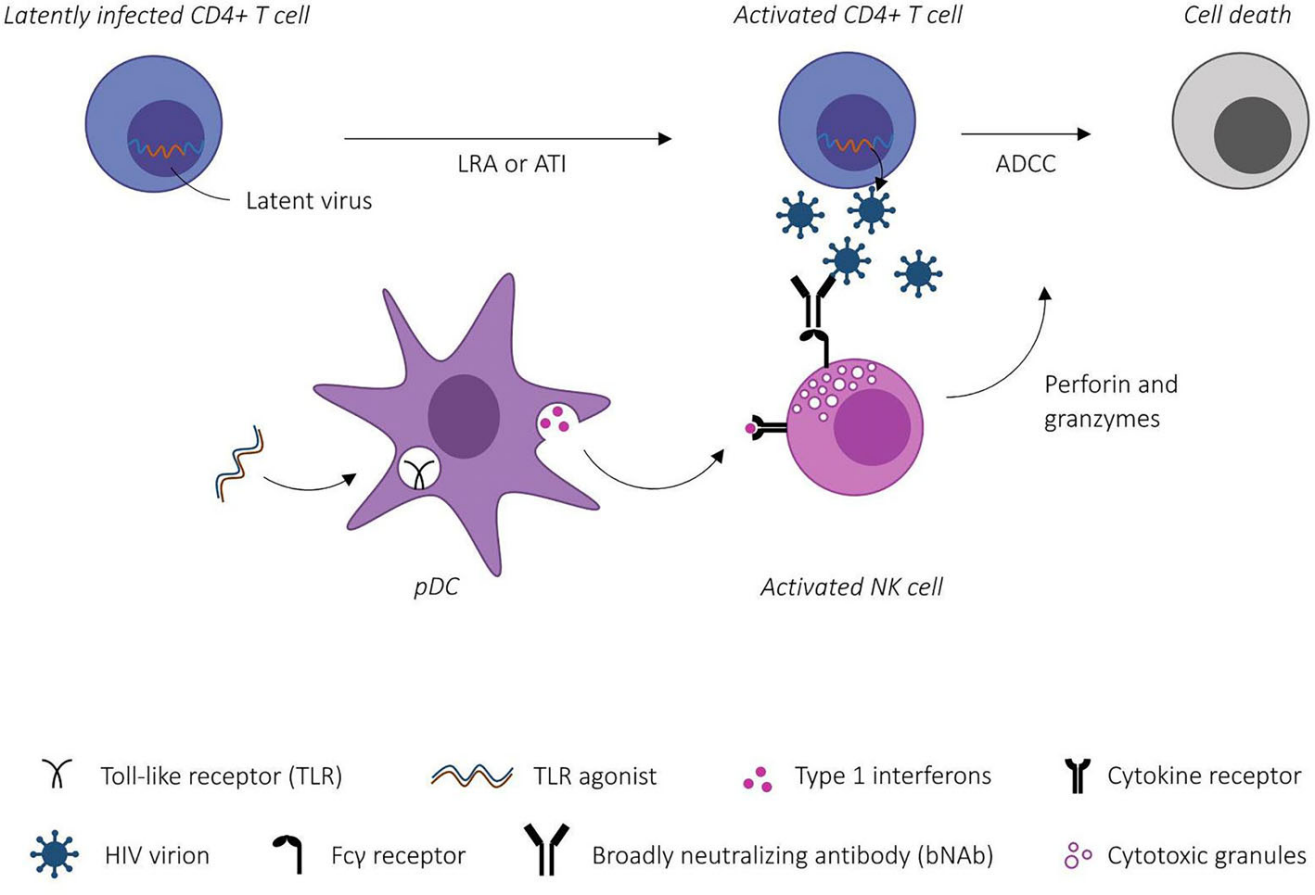
Schematic illustration of Toll-like receptor (TLR)-agonist mediated enhancement of antibody-dependent cellular cytotoxicity (ADCC). TLR-agonists prime the innate immune system through plasmacytoid dendritic cell (pDC) activation. Activated effector cells (here NK cells) bind broadly neutralizing antibody-antigen complexes via Fcγ receptors inducing ADCC-mediated killing of HIV-1 infected CD4+ T cells. [Modified from Martinsen et al. (9)].

Both bNAbs and TLR agonists exhibit advantageous properties towards enhancing the killing of HIV-1 infected cells (see **Figure 1**), however to date only a few studies have investigated the combined effect *in vivo.* Borducchi et al. (19) evaluated the combined effect of the TLR7 agonist GS-9620 and the V3-glycan dependent bNAb PGT121. Fourty-four NHPs were intrarectally challenged with simian-human immunodeficiency virus (SHIV) and treated with ART from day 7 post-infection. Animals were divided in four groups receiving either sham, GS-9620, PGT121 or a combination. ART was continued for 16 weeks after the last dose of PGT121 to allow for plasma levels of antibodies to drop under the previously determined threshold for neutralization-prevented viral rebound (1 μg/mL) (70). 196 days post ATI only six out of 11 NHPs in the combined PGT121+GS-9620 group experienced viral rebound compared to nine out of 11 in the PGT121 group, 10 out of 11 in the GS-9620 group and 11 out of 11 in the sham group. Viral DNA was undetectable in PBMCs in all groups at week 96, presumably due to early initiation of ART. Notably, viral DNA was detected in LNMCs in all groups with lower levels detected in the PGT121+GS-9620 group at week 120 compared to the sham group (P = 0.004). Despite undetectable viral DNA levels in PBMCs, all sham-treated NHPs rebounded, suggesting that the sensitivity of current viral DNA assays is insufficient to predict viral rebound in line with observations from previous clinical case reports (71).

Adoptive transfer of PBMCs and LNMCs from rebounding NHPs to SHIV-naïve animals resulted in infection, whereas adoptive transfer with cells from non-rebounding NHPs were unable to induce infection. Furthermore, depletion of CD8+ T cells and NK cells in non-rebounding NHPs failed to induce plasma viremia. Although it may be concluded that bNAb administration in combination with immune stimulation with the TLR 7 agonist GS-9620 reduced the viral reservoir, it is important to note that the study used an acute seven-day infection model before ART initiation, which allows for preservation of the host immune system and have been shown to result in a relatively small viral reservoir (72–74). The study observed a maximum therapeutic effect in animals with the lowest pre-ART viral loads suggesting that attaining similar results would be more difficult in animals which started ART during chronic infection.

More recently, Hsu et al. (20) investigated the dual effect of the two bNAbs N6-LS and PGT121 targeting different regions of the HIV-1 Env protein in addition to combinatorial treatment with the TLR7 agonist GS-986. 16 rhesus macaques were infected by intrarectal inoculation with SHIV and started a daily ART regimen on day 14 post-infection. Animals were randomly assigned to an active arm receiving the combination of GS-986, N6-LS and PGT121 or a control arm receiving sham. Animals were monitored for the development of anti-drug antibodies (ADA) against either bNAb and if ADA developed treatment with the respective bNAb was suspended. In the active arm ATI was initiated after plasma levels of N6-LS and PGT121 were < 0.25 μg/mL for four weeks, whereas ATI was initiated in all animals in the control arm at week 40.

Consistent with previous studies, GS-986 administration was associated with the induction of peripheral plasma immune activation (17). However, measuring SHIV RNA levels in plasma 24-hours post-GS-986 administration revealed no viral blips. The study observed a strong correlation between SHIV RNA and DNA levels both pre-ART and at the time of ATI suggesting that the level of peak viremia is correlated with the size of the viral reservoir both before and during ART. All animals rebounded, however GS-986+N6-LS+PGT121 treatment was associated with delayed time to viral rebound by two-fold with a median time to rebound of three weeks in the control arm and six weeks in the active arm (P = 0.024). Of note only one animal in the active arm received the full number of bNAb infusions as the remaining animals developed anti-drug antibodies (ADA). However, the animal receiving all bNAb infusions did not experience a longer time to viral rebound when compared to animals that received an incomplete number of infusions. Importantly, this study was able to reproduce findings from Borducchi et al. (19) demonstrating delayed viral rebound after combinatorial treatment with dual bNAbs and a TLR 7 agonist in a different macaque colony using a different SHIV strain but overall, the results were less impressive than those over in the Borducchi paper.

However, the study by Hsu et al. may better reflect a real-life setting where ART initiation is started very early, i.e., day 14 post-inoculation compared to extremely early, i.e., 7 days post-inoculation in the Borducchi study. This would be similar to the situation in the clinical RV254 acute HIV infection cohort in Thailand (75), hereby potentially translating the impact of this experimental treatment to HIV-infected humans more accurately. The Hsu study is however limited by incomplete study infusions of bNAbs due to the development of ADA, hereby risking an underestimation of the potential treatment effect as well as by the variation in ART duration among the two study arms to accommodate bNAb levels < 0.25 μg/mL before ATI initiation.

## Conclusions

The preclinical studies included in this review reported beneficial immune stimulation of innate effector functions and enhanced ADCC elimination of HIV-1 infected cells after intervention with either TLR agonists or bNAbs. Although clinical studies evaluating monotherapy with either agent did not observe sustained viral control after ART cessation, findings in two independent NHP-cohorts suggest that combining TLR agonists and bNAbs may delay viral rebound and potentially lead to long-term remission in the absence of ART. Collectively, the clinical studies included in this review indicate that the combination of bNAbs and TLR agonists are well-tolerated and therefore of particular interest for further investigation in clinical HIV-1 cure trials. However, factors such as immune exhaustion, bNAb sensitivity to distinct viral reservoir profiles and time for intervention might affect the clinical success. Indeed, results from two ongoing clinical trials (NCT03837756, NCT04357821) investigating the combination of a TLR 9 agonist (MGN1703) with two bNAbs (3BNC117+10-1074 or VRC07+10-1074) are expected and will shed more light on the potential value of this combination as a means towards an HIV-1 cure.

## Author Contributions

CH and OS conceived, designed and carried out the project. CH and OS drafted, reviewed and edited the manuscript. All authors contributed to the article and approved the submitted version.

## Funding

This research was supported by The Central Region Denmark Research Fund (grant #7016-00022B) and The Independent Denmark Research Fund (grant #A3233).

## Conflict of Interest

OS has served as a scientific advisor for Abbvie, Gilead, Mologen AG, and Immunocore for work unrelated to this project.

The remaining author declares that the research was conducted in the absence of any commercial or financial relationships that could be construed as a potential conflict of interest.

## Publisher’s Note

All claims expressed in this article are solely those of the authors and do not necessarily represent those of their affiliated organizations, or those of the publisher, the editors and the reviewers. Any product that may be evaluated in this article, or claim that may be made by its manufacturer, is not guaranteed or endorsed by the publisher.
